# Acoustic impedance matched buffers enable separation of bacteria from blood cells at high cell concentrations

**DOI:** 10.1038/s41598-018-25551-0

**Published:** 2018-06-14

**Authors:** Pelle Ohlsson, Klara Petersson, Per Augustsson, Thomas Laurell

**Affiliations:** Departament of Biomedical Engineering, Lund University, Lund, Sweden

## Abstract

Sepsis is a common and often deadly systemic response to an infection, usually caused by bacteria. The gold standard for finding the causing pathogen in a blood sample is blood culture, which may take hours to days. Shortening the time to diagnosis would significantly reduce mortality. To replace the time-consuming blood culture we are developing a method to directly separate bacteria from red and white blood cells to enable faster bacteria identification. The blood cells are moved from the sample flow into a parallel stream using acoustophoresis. Due to their smaller size, the bacteria are not affected by the acoustic field and therefore remain in the blood plasma flow and can be directed to a separate outlet. When optimizing for sample throughput, 1 ml of undiluted whole blood equivalent can be processed within 12.5 min, while maintaining the bacteria recovery at 90% and the blood cell removal above 99%. That makes this the fastest label-free microfluidic continuous flow method per channel to separate bacteria from blood with high bacteria recovery (>80%). The high throughput was achieved by matching the acoustic impedance of the parallel stream to that of the blood sample, to avoid that acoustic forces relocate the fluid streams.

## Introduction

Sepsis is a systemic response to an infection, and the severe form is a common condition with a yearly incidence rate of 0.3% in the US, and mortality as high as 29%^1^. This makes sepsis one of the most common reasons for being admitted to intensive care^2^ and the 10^th^ most common cause of death^3^. Sepsis is most often caused by bacteria, making fast and appropriate antibiotics treatment crucial for patient survival^4,5^.

Treatment is ideally guided by identification of the pathogen and its antibiotic resistance pattern. The gold standard for detection of bacteria in blood samples is blood culture, where the sample commonly is cultured in enriched broth until growth is detected. Bacterial strain and antibiotic resistance is then identified by Gram staining and sub culturing, polymerase chain reaction (PCR)^6^ or mass spectrometry (MS)^7^. Unfortunately the initial culture step may take from hours to days^8^, which is too long since mortality increases with 8% per hour of delay of efficient antimicrobial treatment during the first 6 hours of septic shock^4^. Furthermore, all culture-based detection methods have limited sensitivity, as they may miss slow-growing or fastidious bacteria and bacteria in samples containing antibiotics^9^.

While awaiting identification of the sepsis causing pathogen, the patient is treated with broad-spectrum antibiotics. This initial treatment is often not sufficient and in 47% of the cases the patient does not respond to this treatment at all^10^. Broad-spectrum antibiotics do not only act on the disease-causing bacteria, but also on the patient’s normal bacteria flora, causing additional harm. Furthermore, extensive use of broad spectrum antibiotics drives the increasing antibiotics resistance^11^. A more recently established and fast alternative to blood culture is direct PCR detection without previous culture, typically preceded by lysis of the blood and nucleic acid purification^12^. However, current PCR methods suffer from limited sensitivity and specificity, and susceptibility to contamination^12,13^.

Consequently, there is an urgent need for faster and more sensitive procedures for pathogen identification. An alternative would be to separate the bacteria from the red and white blood cells (henceforth collectively called blood cells) based on differences in physical properties prior to PCR detection. Such a separation is challenging, since samples from septic patients typically contain as little as 1–100 colony forming units (cfu) of bacteria or less^14^ among typically 3∙10^9^ red blood cells (RBCs), 1∙10^7^ white blood cells (WBCs) and 2∙10^8^ platelets per ml of blood^15,16^. To find the few bacteria that are present in a blood sample, it is critical that the separation method has a very high bacteria recovery, efficient blood cell removal and sufficiently high throughput to process a clinical sample within reasonable time. Additionally, if such methods were available, bacteria detection or removal in blood components for transfusion could potentially prevent sepsis caused by contaminated blood components^17^.

Several microfluidic separation methods have been applied to separate bacteria from blood cells^18,19^, including dielectrophoresis^20–22^, inertial effects^23–26^, cell margination^27,28^, surface acoustic waves^29,30^, bead-based extraction^31–33^, filtering^34^, centrifugal microfluidics^19^ and lysis-based methods^35,36^. The suitability of these methods for sample preparation in sepsis diagnosis is, however, limited, either by a too costly labelling, low sample throughput, limited removal efficiency of cells or lysis debris, or too low bacteria recovery.

In this study we address this challenge by separating bacteria from blood cells through bulk acoustophoresis. We separate the smaller bacteria from the larger blood cells based on the principle that the acoustically induced velocity of a suspended object scales with the square of its radius^37,38^. Previously we have shown that it is possible to separate bacteria from patient blood samples using bulk acoustophoresis, followed by acoustic enrichment and PCR detection of the separated bacteria^39–42^. The sensitivity of the method was, however, limited by that only 11% of the bacteria were recovered in the separation step. In this study we have addressed the modest bacteria recovery reported by our group^43^ and later others^44,45^. Using media with adjusted acoustic impedance^46–49^, we can now recover 99.7% of the bacteria while removing more than 99.9% of the blood cells. At higher blood concentrations, 20% whole blood, we can still recover 90% of the bacteria while removing more than 99% of the blood cells enabling 1 ml undiluted whole blood to be processed within 12.5 min. To the best of our knowledge that makes this, the fastest label-free microfluidic continuous flow method per channel to separate bacteria from blood with high bacteria recovery (>80%). Combined with downstream enrichment and detection methods in a similar fashion to what we have previously reported, this paves the way for faster and more automated sepsis diagnostics, which potentially could reduce mortality.

## Separation Considerations

### Separation principle

**Figure 1:**
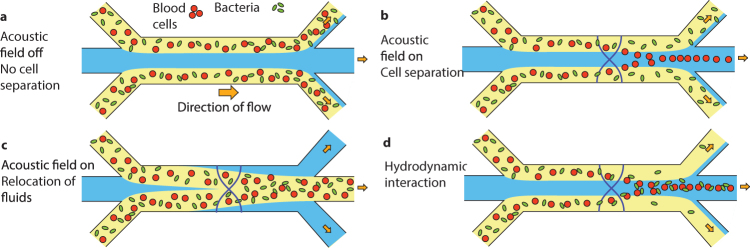
Acoustic separation principle. (**a**) Acoustic field off; sample with blood cells (red) and bacteria (green) in plasma (yellow) laminated along sidewalls by a central cell-free medium (blue). (**b**) Acoustic separation; acoustic field applied, focusing blood cells but not bacteria. (**c**) Acoustic relocation of fluid streams due to acoustic impedance mismatch; the fluid with the highest acoustic impedance is relocated to the central pressure node. (**d**) Hydrodynamic interaction; blood cells pull neighbouring bacteria with them through hydrodynamic drag.

The principle for acoustic separation of bacteria from blood cells is illustrated in Fig. 1. The blood sample, containing bacteria, is laminated on both sides of a central cell-free medium (Fig. 1a). This central flow stream is important to achieve high recovery of bacteria and to enable separation at high blood cell concentrations, because it allocates space for the blood cells to migrate into.

When the ultrasound is turned on, a standing acoustic wave is formed between the side walls of the separation channel, creating a primary radiation force on the blood cells directed towards the pressure node located along the centreline of the channel (Fig. 1b). While flowing through the channel, the blood cells are acoustophoretically transported into the cell-free medium and removed through the centre outlet.

The bacteria, which are an order of magnitude smaller compared to the blood cells, are much less affected by the acoustic radiation force, since the acoustophoretic velocity is proportional to the square of the particle radius^37,38^. In comparison to the blood cells the bacteria are in principle unaffected by the sound field and hence remain in the blood plasma that flow along the side walls to the side channel exits. Any free DNA in the blood plasma is also likely to stay in the plasma and exit with the bacteria. To create some margin to also collect those bacteria that exist at or cross the interface between the laminated liquids, the flow rate of the side outlet was set 50% higher than the side inlet.

### Acoustic field acting on inhomogeneous fluids

The acoustic field does not only create a force on the blood cells but the laminated fluid streams can themselves be affected depending on their relative acoustic impedance (i.e. density times speed of sound). This is analogous to how liquids can be layered in a gravity field based on differences in mass density. In this configuration a fluid with high acoustic impedance laminated along the side walls by a fluid with lower acoustic impedance will relocate to the centre of the channel when exposed to the acoustic field^50,51^.

In the proposed separation the presence of blood cells and plasma proteins in the sample increases the acoustic impedance of the sample stream relative to a regular buffer and hence the sample will relocate to the centre outlet without any separation (Fig. 1c)^49,52^. In the current study the relocation of the sample stream was prevented by increasing the acoustic impedance of the central medium to match the acoustic impedance of 20% whole blood. Supplementary Figure S1 shows the acoustic impedance for blood at different dilution factors ranging from pure buffer to 20% whole blood, in relation to the acoustic impedance of the unmatched and matched buffer.

### Hydrodynamic interactions

Another effect that can hamper the separation is hydrodynamic interaction between the blood cells and the bacteria. Blood cells that are focused to the centre drag along a thin layer of surrounding liquid, including any therein suspended bacteria, to the central stream which interferes with the separation (Fig. 1d). This effect is stronger the higher the blood cell concentration is. Theoretical estimates yield that the hydrodynamic interaction is starting to impact separations at particle volume ratios above 0.001 and at ratios above 0.01 the hydrodynamic particle interaction becomes prominent^53^. A rule of thumb is that as long as the mean particle distance is more than ten times the particle diameter the hydrodynamic interaction can be neglected.

### Acoustic streaming

Apart from creating a force on particles and liquids, the acoustic field can also induce acoustic streaming^54^ in the fluid and this can lead to a transfer of bacteria or molecules across the streamlines^55^. For a given acoustic field this effect is more prominent at low flow velocities due to the longer retention time in the channel. For larger objects such as blood cells, the effect of the radiation force is, however, much larger than the effect of acoustic streaming in our system.

## Materials and Methods

### Microfluidic chip

**Figure 2:**
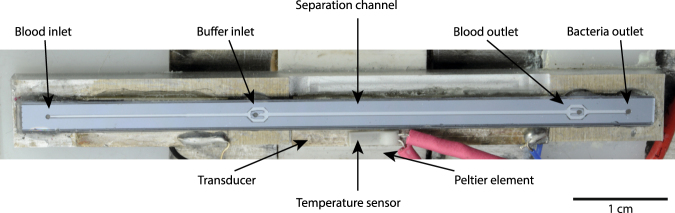
The experiments were performed in a wet etched silicon chip sealed with a glass lid. A piezoelectric transducer generated the acoustic field while a Peltier element held the temperature at 25 °C.

The separation method was tested using a silicon chip with anisotropically wet etched channels sealed by a glass lid, shown in Fig. 2. The chip has a common inlet that branches into a trifurcation with a central buffer inlet that laminates the sample along the separation channel side walls. The separation channel is 29 mm long, 375 µm wide and 150 µm deep and ends with a trifurcation having a central outlet and the side branches connected to a common side outlet. The chip^50^ and fabrication procedure^56^ have been described in more detail previously.

### Acoustic actuation

The chip was acoustically actuated by a piezoelectric transducer glued underneath the separation channel and connected to a waveform generator (Tektronix AFG3022B, UK Ltd., Bracknell, UK) through an in-house built amplifier. The driving voltage was monitored with an oscilloscope (Tektronix TDS2002C, UK Ltd., Bracknell, UK) and held at 15 V peak-to-peak through an in-house written software controlling the waveform generator. The actuation voltage was chosen to be the highest possible where temperature feed-back loop could still retain the chip at 25 °C. The actuation frequency was tuned by visual optimization of the acoustic focusing and was set to 1.99 MHz unless otherwise stated.

### Temperature control

To reach high separation stability over extended time periods, the temperature of the chip was controlled at 25 °C by a Peltier element in contact with the aluminium chip holder. The Peltier element was regulated using a PID regulator (TC0806-RS232 CoolTronic GmBH, Beinwill am See, Switzerland) connected to a Pt1000 temperature sensor glued to the piezoelectric transducer.

### Flow control

**Figure 3:**
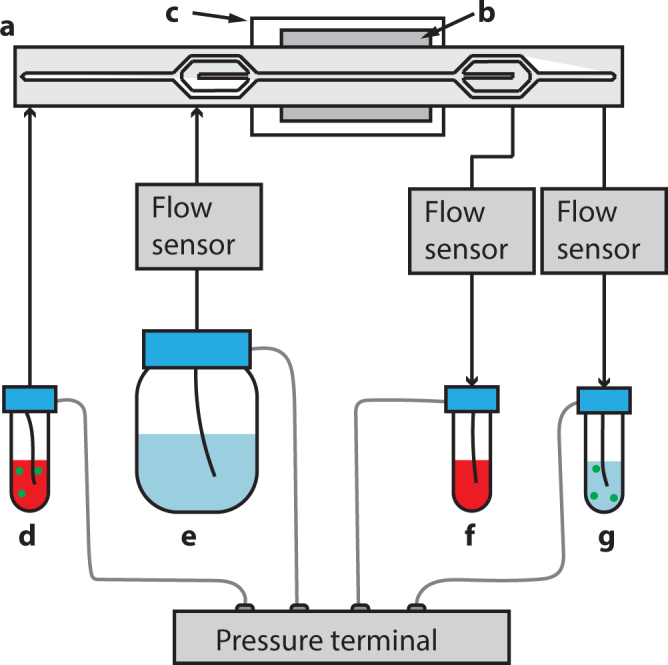
Schematic of the system. The acoustophoresis chip (**a**) is actuated by the piezoelectric transducer (**b**) and the temperature is controlled by a Peltier element (**c**). Sample containing blood and bacteria (**d**) enters the chip through the first inlet. Impedance matched buffer (**e**) enters through the centre inlet. Blood cells focused into the centre buffer exit the chip through the centre outlet and into the waste container (**f**) while bacteria exit through the last outlet and end up in a collection tube (**g**). All flows are controlled by a pressure terminal and flow sensors.

All flows through the chip were regulated by controlling the pressure in sample tubes connected to the side inlet, side outlet and centre outlet as well as a buffer bottle connected to the centre inlet using a pressure terminal (Fig. 3; VEMA, FESTO, Germany). The pressures were PID regulated through in-house developed LabView software (National Instruments Corporation, Austin, Texas, USA) based on flow rates measured by thermal flow sensors (SLI-1000, Sensirion AG, Staefa ZH, Switzerland). The flow control system has been further described previously^50^.

### Bacteria culture and staining

*Pseudomonas putida* KT2440 (Trevisan) Migula (ATCC® 47054™) was chosen as a biosafety level 1 model for the common sepsis pathogen *Pseudomonas aeruginosa*^14^. *P. putida* were cultured overnight on a shaker at 30 °C in medium mixed from tryptone (30 g/l), yeast extract (20 g/l) and 3-(N-Morpholino)propanesulfonic acid (MOPS, 10 g/l), adjusted to pH 7 and sterilized by autoclaving. Before addition to the blood sample they were washed by centrifugation and stained with SYTO® 9 green fluorescent nucleic acid stain (Life Technologies, Grand Island, NY, USA).

### Blood collection and dilution

Venous peripheral blood samples were collected from healthy volunteers in Vacutainer® tubes either containing sodium citrate solution (10% of final volume) or spray-coated with ethylenediaminetetraacetic acid potassium salt (K_2_EDTA) as anticoagulant (BD Biosciences, Franklin Lakes, NJ, USA). All samples were diluted in 1× phosphate-buffered saline (PBS) containing 0.05% sodium azide (NaN_3_) to prevent the added bacteria from multiplying and 2 g/l bovine serum albumin (BSA) to avoid cell adhesion to the channel walls (further on referred to as buffer). The study protocol was approved by The Regional Ethical Review Board in Lund (Dnr 2011/400) and informed consent was obtained from all donors. All methods were carried out in accordance with relevant guidelines and regulations.

### Cell counting

**Figure 4:**
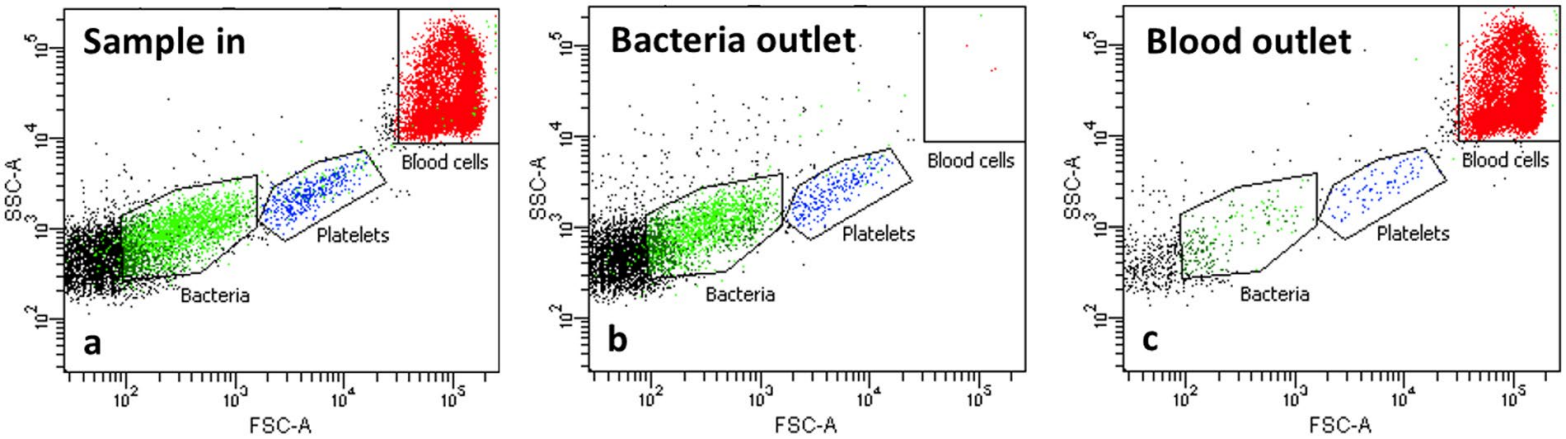
Flow cytometry analysis. Input sample (**a**) and samples from bacteria (**b**) and blood (**c**) outlets analysed by flow cytometry. Blood cells (red) and platelets (blue) are gated on forward scatter (FSC-A) and side scatter (SSC-A), while bacteria (green) are gated on fluorescence (not shown).

All cells were counted through flow cytometry (FACSCanto II cytometer and FACSDiva software, BD Biosciences, Franklin Lakes, NJ, USA), as exemplified in Fig. 4. The analyzed sample volume was measured using counting beads (Trucount™ tubes, BD Biosciences, Franklin Lakes, NJ, USA; Bacteria Counting Kit, Life Technologies, Grand Island, NY, USA). The RBC, WBC and platelet populations were initially identified through CD235a, CD45 and CD41a antibody labelling respectively (BD Biosciences, Franklin Lakes, NJ, USA) and subsequently gated through forward (FSC) and side scatter (SSC) (Fig. 4). The RBC and WBC populations overlap in forward and side scatter and were therefore counted together as blood cells. Most of the cells that are counted as blood cells are RBCs since they are typically 2-3 orders of magnitude more abundant than the WBCs, therefore this number is also a good estimate of the number of RBCs. The bacteria were gated on fluorescence, since they otherwise would overlap with background noise and the platelet population in forward and side scatter.

The bacteria recovery is here estimated as the fraction of the bacteria exiting the system through the side outlet relative all bacteria exiting side and centre outlet. The blood cell removal efficiency is the number of blood cells exiting through the centre outlet relative the total number of blood cells exiting the system. All reported numbers are average steady state separation performance corrected for the volume of buffer present in the system at the start of a run and the volume of sample left in the system at the end of a run.

### Visual confirmation of the bacteria separation principle

The separation principle was confirmed by visual inspection of the inlet and outlet trifurcations through a microscope. Citrated blood was diluted to 1% (0.9% blood when the citrate solution is accounted for) in buffer and injected at 200 µl/min through the sample inlet. To visualize the plasma flow in the chip the sample was spiked with 1 g/l fluorescein sodium (The British Drug Houses LTD., Poole, England) instead of bacteria. The plasma was laminated along the side walls by a central buffer flow (300 µl/min) containing 1% Percoll (Sigma-Aldrich, Schnelldorf, Germany) to increase the acoustic impedance of the central flow stream. The outlet flow rates were set to 300 µl/min for the side outlet and 200 µl/min at the centre outlet and the chip was actuated in a half wavelength resonance mode at 1.989 MHz.

### Optimization and evaluation of the bacteria separation

The separation performance was optimized and evaluated using diluted whole blood spiked with *P. putida* bacteria. Whole blood with EDTA as anticoagulant was diluted in buffer and spiked with 5∙10^5^ *P. putida* bacteria per µl whole blood. This high concentration of bacteria was used to enable evaluation of the acoustic separation process by counting individual bacteria in the respective outlets using flow cytometry. The bacteria concentration was still considered low enough to prevent the bacteria concentration from affecting the separation.

To test the influence of blood concentration on the bacteria separation, blood samples were diluted to 1, 2, 5, 10 and 20% whole blood. Flow rates were 200 µl/min sample input, 300 µl/min centre buffer, 300 µl/min exiting through the joint side outlet and 200 µl/min exiting through the centre outlet. To demonstrate the importance of acoustic impedance matching of the laminated fluids, either a pure buffer or an impedance matched buffer (70% buffer and 30% Histopaque 1077; Sigma-Aldrich, Schnelldorf, Germany) was used as centre buffer.

The effect of varying the flow rate was subsequently tested by performing the same experiment at sample flow rates of 50, 100, 200 and 400 µl/min for 1 and 20% whole blood. The ratios between all flow rates were kept the same as in the previous measurements. In these experiments an impedance matched centre buffer consisting of 70% buffer and 30% Histopaque 1077 was used.

### Data availability

All data generated during the current study is available from the corresponding author on request.

## Results and Discussion

### Visual confirmation of the bacteria separation principle

**Figure 5:**
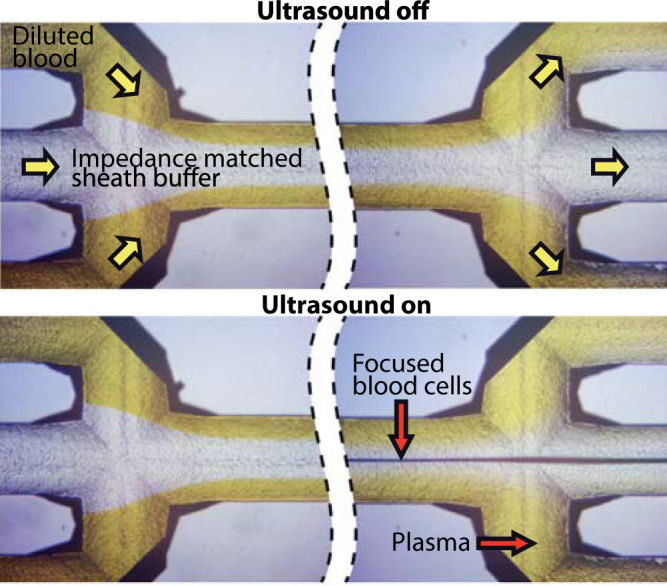
Microscope images of the chip inlet and outlet trifurcations with ultrasound off and on. The blood sample enters through the side channels and is laminated along the side walls. When the ultrasound is turned on, blood cells are acoustically focused from the plasma to the centre buffer and removed through the centre channel. The plasma, spiked with yellow dye for visualization, remains laminated along the side walls and is collected through the side channels.

The separation principle was confirmed visually through microscopy of the inlet and outlet trifurcations with the ultrasound turned off and on (Fig. 5). A blood sample diluted to 0.9% was injected through the sample inlet and was laminated on either side of an impedance matched cell free medium injected in the centre inlet. When the ultrasound was turned on, the blood cells were tightly focused by the primary acoustic radiation force to a narrow dark line in the centre buffer and removed through the centre outlet. The plasma, here visualized with dye, remained near the side walls and exited through the side channels to the bacteria outlet. A narrow band of clean buffer was seen in the outlet side branches, creating some margin for recovering also slightly displaced bacteria. This margin was intentionally created by setting the centre inlet buffer flow rate higher than the centre blood outlet flow rate. The slight broadening of the plasma stream at the outlet (Fig. 5 lower right) when the ultrasound is active as compared to without ultrasound is attributed to the effect of acoustic streaming.

### Acoustic impedance matching and hydrodynamic interaction

Performing the separation at high cell concentrations increases throughput and reduces separation time. However, elevated cell concentrations can compromise separation performance due to increased hydrodynamic cell-bacteria interactions. Increasing the blood concentration also increases the acoustic impedance of the sample, which may cause sample relocation to the centre hindering separation if the acoustic impedance of the sample increases above that of the centre buffer.

**Figure 6:**
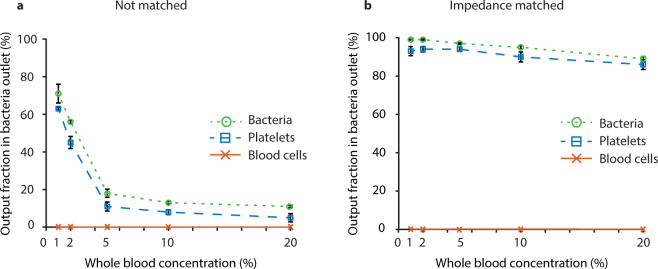
Effect of impedance matching and blood concentration on separation performance. Fraction of total output of each particle type in the bacteria outlet for 1, 2, 5, 10 and 20% blood without (**a**) and with (**b**) acoustic impedance matching of the centre buffer at a sample flow rate of 200 µl/min. The plots show mean values ± standard deviation of three repeats.

To investigate critical boundaries with respect to blood concentration the recovery of bacteria and the removal efficiency of blood cells were measured for whole blood concentrations ranging from 1–20% at a sample flow rate of 200 µl/min. The separations were performed with (Fig. 6b) and without (Fig. 6a) acoustic impedance matched medium as the central buffer. Higher whole blood concentrations than 20% were not possible since that did compromise flow sensor accuracy.

The blood cells were efficiently removed in all experiments, with less than 0.2% exiting through the bacteria outlet for all experiments (Fig. 6a,b, red lines). The bacteria recovery, on the other hand, was highly dependent on acoustic impedance matched centre buffer (green lines). With standard cell-free medium (Fig. 6a) a bacteria recovery of 71% could be achieved for 1% blood, but this quickly dropped to below 20% for blood concentrations of 5% and above. The reduced bacteria recovery at higher whole blood concentrations is due to the increased acoustic impedance caused by the high cell and plasma concentration of the sample stream in relation to the cell-free central medium. This causes the whole sample liquid, including both bacteria and blood cells, to relocate to the centre of the channel and exit through the blood outlet.

On the contrary, Fig. 6b demonstrates that the sample relocation does not occur when increasing the acoustic impedance of the central cell-free medium. With high impedance cell-free medium, the bacteria recovery was as high as 99% for 1% whole blood and dropped to 89% for 20% whole blood. The drop in recovery can be explained by an increased hydrodynamic interaction causing the blood cells to drag bacteria into the central stream. At 20% whole blood the cell volume fraction is approximately 0.08–0.1 (assuming an haematocrit of 40–50%), which vastly supersedes the critical level where hydrodynamic interaction starts to impact the acoustophoretic separation^53^.

The data underpins the importance of acoustic impedance matching of the central cell-free medium to achieve efficient separation, but also demonstrates that in spite of the negative influence of the hydrodynamic interaction at high blood concentrations (20% whole blood) a relatively high bacteria recovery of 89% can be accomplished, which may be sufficient when high throughput is priority and larger sample volumes are available.

### Optimization of flow rates

**Figure 7:**
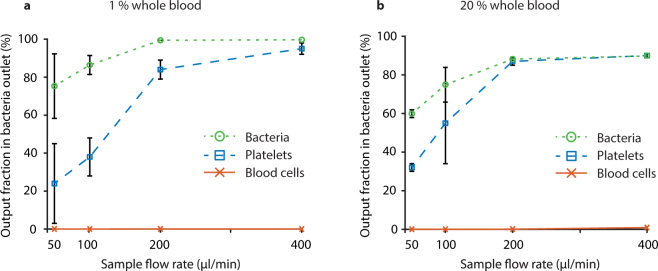
Effect of blood concentration and flow rate on separation performance. Fraction of total output of each particle type in the bacteria outlet for different flow rates when running **(a)** 1% whole blood and **(b)** 20% whole blood. The plots show mean values ± standard deviation of three repeats. The centre buffer was impedance matched using 30% Histopaque.

To maximize throughput, sample flow rates of 50–400 µl/min were tested for 1% and 20% whole blood (Fig. 7a,b, respectively) using a central impedance matched buffer. These flow rates were chosen to stay within the range of the flow sensors. Even at high flow rates, the passage time for blood cells through the separation channel was sufficient for efficient focusing and removal. The blood cell contamination in the bacteria outlet remained below 0.1% for all settings except at the highest flow rate for 20% whole blood, where it was 0.87%.

For both blood concentrations, the bacteria recovery was improved at higher flow rates, reaching 99.7% for the 1% blood processed at maximum flow rate. This is because the bacteria spend less time in the separation channel at higher flow rates and are thus less affected by the acoustic radiation force and streaming that may otherwise mix them into the central fraction. The bacteria recovery is higher for the lower blood concentration due to lower hydrodynamic interaction, which is consistent with the data in Fig. 6.

### Platelet separation

Platelets are only slightly larger than the bacteria that were used in these experiments and as expected they behaved similarly in the separations (blue lines in Figs 6–7). The presented separation method can therefore also be used to separate platelets from blood cells with 95% of the platelets exiting through the side outlet and 99.9% of the blood cells exiting through the centre outlet (Fig. 7a). This can be compared to earlier data from Dykes *et al*.^57^ for removal of platelets from peripheral blood progenitor cell products in a similar setup, where 89% of the platelets exited through the side outlet and 97.8% of white blood cells exited through a central outlet. More recent data from Chen *et al*.^49^ demonstrates separation of platelets from blood at an impressive throughput of 5 ml/min, but at a modest separation performance of 88.4% RBC removal and a platelet recovery of 86.2% in a large 17 mm wide layered acoustic resonator with impedance matched buffers.

Since the platelets are slightly larger than the bacteria, they display somewhat higher acoustophoretic mobility. The difference in focusing between platelets and bacteria is most apparent at low flow rates for 1% blood (Fig. 7a), and the gap indicates that also the platelets can be acoustically separated from the bacteria if desired by optimizing the separation further.

### Optimized separation settings

Based on the results above, various separation settings can be chosen depending on if bacteria recovery, blood cell removal efficiency or sample throughput is prioritized (Table 1, priority in bold face). The highest bacteria recovery that was achieved was 99.7%, while the highest blood cell removal was 99.99%. The shortest time to process 1 ml of whole blood (before dilution 5×) was 12.5 min, while achieving a bacteria recovery of 90% and a blood cell removal efficiency above 99%.

**Table 1:** Comparison of label-free microfluidic continuous flow methods for separation of bacteria from red blood cells.

Method	Bacteria recovery	RBC removal	Bacteria enrichment relative RBCs	Sample flow rate	Sample dilution factor	Time to process 1 ml undiluted blood	Comments
Dielectrophoresis^20^	87.2%	100%	∞	0.5 µl/min	20	28 days	
Inertial lift forces^23^	>80%	90%	>8	200 µl/min	200	17 h	
Soft inertial force^24^	N/A	N/A	N/A	18 µl/min	22	20 h	
Elasto-inertial^26^	76%	92%	9.5	0.5 µl/min	1	1.4 days	RBC removal estimated by WBC focusing
	73%	71%	2.5	1 µl/min	1	17 h	
Margination^27^	80%	83.2%	5	16.7 µl/min	1	1 h	
Margination^28^	~70%	~90%	~7	30 µl/min	1	33 min	Single channel device
	~50–60%	N/A	N/A	N/A	1	N/A	16 channel device
	N/A	N/A	N/A	78 µl/min	1	12.8 min	32 channel device
SAW^30^	88%	99.6%	204	1 µl/min	50	35 days	Assuming no cells lost in system
Acoustophoresis^44^	~10%	99.8%	50	10 µl/min	2.2	4 h	
Acoustophoresis^42^	11%	99.9%	101	80 µl/min	1.11	14 min	
Acoustophoresis^45^	34%	98%	17	110 µl/min	9	1.4 h	Calculated for 45% haematocrit
**Impedance matched acoustophoresis (this work)**	**99.7%**	>99.9%	1108	400 µl/min	100	4 h	**Optimized for bacteria recovery**
	75%	**99.99%**	**5054**	100 µl/min	5	50 min	**Optimized for blood cell removal**
	90%	>99%	103	400 µl/min	5	**12.5 min**	**Optimized for throughput**

All numbers are per channel.

### Comparison to other methods

In Table 1 the results presented in this article are compared to other label-free microfluidic continuous flow methods to separate bacteria from blood. It should be noted that not all methods presented in Table 1 are aiming towards sepsis diagnostics, but should rather be seen as typical data for various microfluidic separation methods.

If the intention is to use the method for sample preparation for sepsis diagnostics, the challenge is typically to find as few as 1–100 cfu, or less, of bacteria^14^ among approximately 3∙10^9^ RBCs, 1∙10^7^ WBCs and 2∙10^8^ platelets per ml of blood^15,16^. To be able to detect any of the few bacteria present in the sample, it is crucial to minimize the bacteria loss in the separation. Inertial, margination and other acoustic methods typically report 20–90% loss of bacteria, which significantly affects sensitivity. Impedance matched acoustophoresis is the method that demonstrates the highest bacteria recovery with down to 0.3% bacteria loss, compared to SAW and dielectrophoresis, the second and third best methods in Table 1, with 12 and 13% bacteria loss respectively.

To be able to concentrate the few bacteria from a large sample to a small detection volume it is necessary to remove the RBCs that typically constitute almost half of the blood volume. Every percent of RBCs that remains add 3∙10^7^ RBCs per cfu of bacteria in a typical sample (1 cfu of bacteria and 3∙10^9^ RBCs per ml), meaning that the removal efficiency should ideally be extremely high. Inertial and margination methods leave 10–29% of the RBCs in the sample, taking up a large volume hindering enrichment and hiding the bacteria among a vast number of cells. In this regard, dielectrophoresis, SAW and acoustophoresis stand out from the other methods in Table 1 as the only methods reaching above 99% RBC removal.

Another key factor for sample preparation is throughput. The low bacteria concentration means that a relatively large sample, often 1–10 ml, has to be processed to enable detection of bacteria. Since every hour is critical in sepsis diagnostics, such a volume should ideally be processed within minutes.

The throughputs of most microfluidic methods such as dielectrophoresis, inertial separation and SAW are hampered by low sample flow rates or high sample dilution factors. The throughput of margination is relatively high since whole blood can be processed, but comes at the cost of low bacteria recovery and RBC removal. However, by using a moderate dilution (5×) and relatively high flow rate (400 µl/min), 1 ml whole blood equivalent can be processed by impedance matched acoustophoresis within 12.5 minutes (~7∙10^6^ RBCs/s), making this the fastest label-free microfluidic continuous flow method per channel achieving high bacteria recovery (>80%).

### Outlook

Although the presented method yields high bacteria recovery, blood cell removal efficiency and throughput, there is still room for further improvement. The currently used setup was limited in flow rate and blood concentration by the flow sensors. Further increase in throughput may be achieved by using higher blood cell concentrations, larger channels^58^ or multiple parallel separation channels^22^. Using higher blood cell concentrations would also reduce the need for subsequent sample concentration, but would demand alternative flow control methods and likely reduces bacteria recovery.

It will be important to confirm this proof-of-concept with a larger number of samples from healthy volunteers as well as septic patients to ensure statistical certainty and robustness to variations in the sample composition. Sepsis may for instance affect RBC size^15^, WBC count^16^ and plasma viscosity^59^, which may in turn affect the separation. Our previous study where 57 blood samples from sepsis patients were processed by acoustophoresis indicated increased RBC sedimentation rate as well as increased platelet activation in some patient samples compared to blood from healthy donors, but without any evidence of detrimental impact on the acoustophoretic separation^42^.

Additionally, the bacteria concentrations are much lower in samples from septic patients than in the current experiments with spiked blood^14^. This is not expected to have affected the separations, since the bacteria were spiked into the blood at approximately ten times lower concentration than the much larger RBCs^16^. The RBCs should consequently dominate any particle-particle interaction, but this should be confirmed. Our previous study did, however, show that acoustic separation, acoustic enrichment and bacteria detection is possible at clinically relevant bacteria concentrations^42^.

The method also needs to be validated for the wide range of bacteria species and strains that may be found in samples from septic patients^14^. They are all similar in size to the model strain and single bacteria are therefore likely to behave similarly in the acoustic separation. A common challenge for all separation based methods is, however, that many pathogenic bacteria are known to form clusters^60^, reside inside WBCs, or adhere to blood cells^61^ or platelets^62,63^. They may then be removed with the larger cells and lost. This problem is shared with all other methods in Table 1 where separation is to some degree based on cell size. Adhesion of bacteria to cells may at least to some extent be remedied by chemically preventing adhesion^64,65^.

The presented acoustophoretic bacteria separation method opens up a route for development of rapid sepsis diagnostics by integrating the acoustic separation with downstream enrichment, purification and detection of the bacteria. Apart from bulk enrichment methods such as centrifugation, more integration-friendly label-free microfluidic enrichment methods including dielectrophoresis^20^, acoustic focusing^55^ and acoustic trapping^66^ have shown promising results. When choosing from these, high bacteria recovery and throughput matched to the separation are key requirements. There are also several possible detection methods, such as microscopy, flow cytometry, mass spectrometry and PCR, with PCR being a particularly strong candidate for detection of low numbers of bacteria including determination of species and antibiotic resistance pattern.

**Figure 8:**
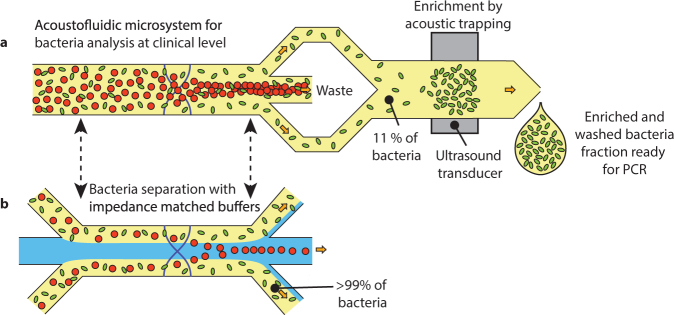
Suggested application of impedance matched acoustic bacteria separation for sepsis diagnostics. (**a**) Previously presented method to acoustically remove red blood cells, acoustically trap bacteria from remaining plasma and finally detect bacteria by PCR^42^ (**b**) Suggested replacement of the initial acoustophoretic blood cell separation with the impedance matched buffer method proposed herein, improving bacteria recovery from 11% to >99%.

We have earlier investigated the possibility to integrate an early version of the acoustic separation with bacteria enrichment through acoustic trapping^66^ followed by chip-based PCR detection as schematically shown in Fig. 8a ^42^. With that prototype system *Escherichia coli* could be detected in the clinical sepsis samples with the highest bacteria concentrations within two hours, underlining both the potential of acoustic sample preparation for sepsis diagnostics as well as the necessity of high bacteria recovery. Replacing the initial acoustophoretic blood cell removal unit with the herein proposed method would increase the demands on subsequent bacteria enrichment because of higher dilution and flow rates, but the lower blood cell contamination and 7–9 times higher bacteria recovery may enable faster sepsis diagnostics also for patients with lower grade bacteremia.

## Conclusions

In this study we have demonstrated that bulk acoustophoresis can be used to separate bacteria from blood cells with, to the best of our knowledge, higher bacteria recovery and throughput per channel than any other label-free microfluidic continuous flow method with high bacteria recovery (>80%) and throughput relevant for diagnostic purposes. This was enabled primarily by matching the acoustic impedance of the centre sheath fluid to the sample, as well as by optimizing the blood concentration and sample flow rate. In combination with downstream bacteria enrichment and detection, the proposed acoustophoresis based bacteria separation method opens up for potential development of new and faster methods to diagnose sepsis.

## Electronic supplementary material

Supplementary Information
