# Supplementary material for: Acoustic impedance matched buffers enable separation of bacteria from blood cells at high cell concentrations
Source: Sci Rep. 2018 Jun 14;8:9156. doi: 10.1038/s41598-018-25551-0 (PMC6002537; doi:10.1038/s41598-018-25551-0)
Supplement: Supplementary file 1 — Supplementary Information [file 41598_2018_25551_MOESM1_ESM.pdf]

Supplementary Information for

Acoustic impedance matched buffers enable separation of bacteria from blood cells at high cell concentrations

Pelle Ohlsson* **, Klara Petersson, Per Augustsson and Thomas Laurell***

Dept. of Biomedical Engineering, Lund University, Lund, Sweden

*Corresponding authors (email: pelle.ohlsson@bme.lth.se, thomas.laurell@bme.lth.se)

** Shared first authorship

Acoustic impedance of samples and buffers

Introduction. A liquid with higher acoustic impedance (density times speed of sound) will be acoustically focused to the pressure node in the center of the channel[1,2]. It is therefore important for an efficient separation that the impedance of the sample is lower than that of the center buffer[3,4].

Methods. The acoustic impedance for the unmatched buffer and matched buffer (70% buffer and 30% Histopaque 1077) were calculated by measuring the speed of sound and density at 25°C in a density and sound velocity meter (Anton Paar DSA 5000 M).

The maximum density and speed of sound for the samples were estimated as the weighted average between the buffer properties and literature values for the density[5] and speed of sound[6] for blood with 60% hematocrit. They were then multiplied to estimate the maximum acoustic impedance of the sample for each blood dilution.

Results. The values found for the density, speed of sound and acoustic impedance are shown in Supplementary Fig. S1 and Supplementary Table S1.

Discussion. The data shows that any addition of blood to the unmatched buffer will increase the acoustic impedance of the sample above the impedance of an unmatched center buffer, leading to relocation of the liquids. It also shows that the acoustic impedance of the diluted blood sample never exceeds that of the matched buffer, even for an extremely high hematocrit such as 60%. This should efficiently prevent liquid relocation.

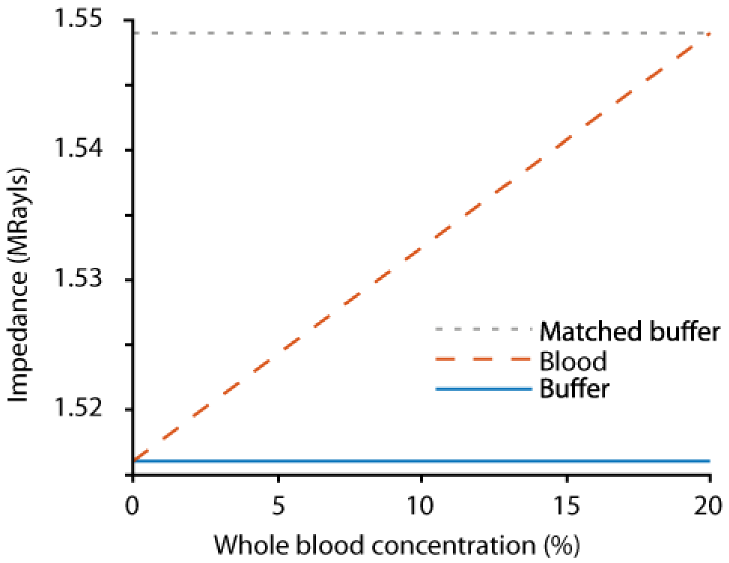

Supplementary Figure S1. Acoustic impedance of buffers and samples. The horizontal lines show the measured values of the acoustic impedance for the unmatched (blue) and matched (grey) buffer respectively. The red line shows the calculated value for blood with high hematocrit (60%, which gives high acoustic impedance) for various dilutions in unmatched buffer, showing that the sample impedance does not exceed the matched buffer impedance.

Supplementary Table S1. Density and speed of sound measured for unmatched and matched buffer (mean and standard deviation of three repeats) and literature values for blood with high hematocrit (60%), as well as calculated acoustic impedance (density times speed of sound).

	Density (kg/m^3)	Speed of sound (m/s)	Acoustic impedance (MRayls)
**Unmatched buffer**	1005.3±0.3	1508.12±0.03	1.5161
**Matched buffer**	1026.6±0.1	1508.94±0.13	1.5490
**Blood (60% hematocrit)**	1064[5]	1583[6]	1.6843

References

1 Deshmukh, S., Brzozka, Z., Laurell, T. & Augustsson, P. Acoustic radiation forces at liquid interfaces impact the performance of acoustophoresis. *Lab on a chip* **14**, 3394-3400, doi:10.1039/c4lc00572d (2014).
2 Karlsen, J. T., Augustsson, P. & Bruus, H. Acoustic Force Density Acting on Inhomogeneous Fluids in Acoustic Fields. *Phys Rev Lett* **117**, 114504, doi:10.1103/PhysRevLett.117.114504 (2016).
3 Chen, Y. *et al.* High-throughput acoustic separation of platelets from whole blood. *Lab on a chip* **16**, 3466-3472, doi:10.1039/c6lc00682e (2016).
4 Tenje, M. *et al.* Acoustophoretic removal of proteins from blood components. *Biomed Microdevices* **17**, 95, doi:10.1007/s10544-015-0003-5 (2015).
5 Kenner, T. The measurement of blood density and its meaning. *Basic research in cardiology* **84**, 111-124 (1989).
6 Bradley, E. & Sacerio, J. The velocity of ultrasound in human blood under varying physiologic parameters. *Journal of Surgical Research* **12**, 290-297 (1972).
